# Shifting hospital care to primary care: An evaluation of cardiology care in a primary care setting in the Netherlands

**DOI:** 10.1186/s12875-018-0734-5

**Published:** 2018-05-09

**Authors:** Tessa C. C. Quanjel, Jeroen N. Struijs, Marieke D. Spreeuwenberg, Caroline A. Baan, Dirk Ruwaard

**Affiliations:** 1 Department of Health Services Research, Care and Public Health Research Institute, Faculty of Health Medicine and Life Sciences, Maastricht University, Maastricht, The Netherlands; 2 Department for Quality of Care and Health Economics, Centre for Nutrition, Prevention and Health Services, National Institute for Public Health and the Environment, Bilthoven, The Netherlands; 3 Research Centre for Technology in Care, Zuyd University of Applied Sciences, Heerlen, The Netherlands; 4 Scientific Centre for Transformation in Care and Welfare (Tranzo), University of Tilburg, Tilburg, The Netherlands

**Keywords:** Substitution, Primary care, Referrals, Hospital care

## Abstract

**Background:**

In an attempt to deal with the pressures on the healthcare system and to guarantee sustainability, changes are needed. This study is focused on a cardiology Primary Care Plus intervention in which cardiologists provide consultations with patients in a primary care setting in order to prevent unnecessary referrals to the hospital. This study explores which patients with non-acute and low-complexity cardiology-related health complaints should be excluded from Primary Care Plus and referred directly to specialist care in the hospital.

**Methods:**

This is a retrospective observational study based on quantitative data. Data collected between January 1 and December 31, 2015 were extracted from the electronic medical record system. Logistic regression analyses were used to select patient groups that should be excluded from referral to Primary Care Plus.

**Results:**

In total, 1525 patients were included in the analyses. Results showed that male patients, older patients, those with the referral indication ‘Stable Angina Pectoris’ or ‘Dyspnoea’ and patients whose reason for referral was ‘To confirm disease’ or ‘Screening of unclear pathology’ had a significantly higher probability of being referred to hospital care after Primary Care Plus.

**Conclusions:**

To achieve efficiency one should exclude patient groups with a significantly higher probability of being referred to hospital care after Primary Care Plus.

**Trial registration number:**

NTR6629 (Data registered: 25–08-2017) (registered retrospectively).

## Background

In an attempt to rein in rising healthcare costs, policy reforms have altered healthcare systems in many Western countries [1, 2]. A considerable number of these reforms focus on limiting the volume growth of more costly hospital care by stimulating substitution of care [3–5]. Substitution of care can be defined as the continual regrouping of resources across and within care settings to exploit the best and least costly solutions in the face of changing needs and demands [6, 7]. Internationally well-known concepts related to substitution such as joint consultations and specialist outreach services concentrate on providing specialist services in primary care settings, improving access to specialist services to enhance the efficiency and quality of health care, and reduce referrals to specialist services in hospital outpatient departments [8–14]. These concepts aim to integrate care by intensifying collaboration and communication between specialists and general practitioners (GP). Previous studies have shown that these kinds of interventions could result in improved patient satisfaction, improved access to specialist care, fewer diagnostic actions and reduced referrals to hospital care [8–11, 13]. Despite these positive findings some studies show that specialist outreach services could lead to higher healthcare costs [12–14].

Also, in the Netherlands, the healthcare sector is focused on substitution of care. For example, to limit the volume growth of more costly specialised care in the hospital setting, all involved healthcare providers have agreed upon shifting less complex treatments from hospital care to primary care [4, 15]. Consequently, several pioneering regions started experiments with a concept called Primary Care Plus (PC+). PC+ is concentrated on the substitution of specialist care in the hospital setting with specialist care in the primary care setting. As GPs act as gatekeepers of the Dutch healthcare system, secondary care provided by specialists is only accessible through GP referral [3]. PC+ initiatives strengthen the gatekeeping and coordinating role of the GP by intensifying collaboration and communication between GPs and specialists. Besides intensifying collaboration between the primary and secondary care sector, PC+ is designed to enhance the connectivity and alignment of care between these sectors [16]. Moreover, it is aimed at improving the health of the population and patients’ experience of care, while at the same time reducing the number of unnecessary referrals to hospital care in order to reduce medical spending [17].

Previous research on PC+ has shown that it is essential to select an appropriate patient population to achieve efficient substitution of care [18]. It should not be designed as an intermediate station between the GP and the hospital. To achieve efficiency it is a precondition that PC+ interventions should exclude patients who need hospital care anyway.

In this study, we focus on a cardiology PC+ centre. Currently, GPs can refer all non-acute and low-complexity patients with cardiology-related complaints to this PC+ centre. Based on certain cardiology-related referral indications and reasons for referral the GP refers a patient to the cardiologist at the centre. After the consultation of a patient, the cardiologist provides the GP a comprehensive description of the results of the test diagnosis and his recommendation regarding further treatment (if needed). This study intends to explore which cardiology-related referral indications and reasons for referral are appropriate for PC+ and which are more appropriate for immediate specialist care in the hospital setting. Hence, patient groups that are more often referred to hospital care after a consultation at the PC+ centre are assumed to be less appropriate for PC+ and preferably should be referred directly to hospital care. The research question is as follows: ‘*Which patients with non-acute and low-complexity cardiology-related health complaints should be excluded from Primary Care Plus and referred directly to specialist care in the hospital?*’

## Methods

### Design

This is a retrospective observational study based on register data. It is part of an extensive practice-based, mixed-methods study focusing on the effects of a cardiology PC+ intervention. The study is approved by the Medical Research and Ethics Committee of the Maastricht University Medical Centre (METC 15–4-032).

### Setting

This study was conducted in a geographically demarcated region located in the most southern part of the Netherlands. The region consists of 11 municipalities, covering 277,000 residents, approximately 135 GPs and one general hospital. Compared to the overall population of the Netherlands, the region is characterised by a relatively unhealthy population with a low socio-economic status [19, 20]. In 2013, the Minister of Health appointed nine regions in the Netherlands as so-called pioneer sites aimed at restructuring health services based on population management. This means focusing on addressing health needs at all points along the continuum of health and well-being for a specified population by integrating services across health care, prevention, social care and welfare [21, 22]. This study is focused on the pioneer site called MyCare. MyCare is a regional partnership between the care group (a legal entity exclusively owned by GPs), the hospital, the patient representative foundation and the dominant health insurance company in the region, CZ. One of the interventions of MyCare is the cardiology PC+ centre.

### Intervention

**Fig. 1:**
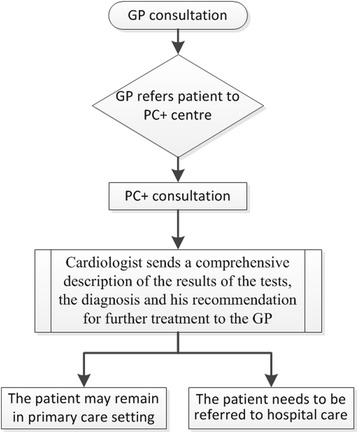
The flow of patients

In the cardiology PC+ centre cardiologists provide consultations in a primary care setting. All GPs in the region are able to refer non-acute and low-complexity patients with cardiology-related complaints to the centre. A consultation at the centre consists of the following diagnostic tests: a blood test, an ECG, an echo and an exercise test. After the tests, patients meet the cardiologist, who explains the results of the tests. The cardiologist sends a comprehensive description of the results of the tests, the diagnosis and his recommendation regarding further treatment (if needed) to the GP. The two overall recommendations are: 1) the patient may remain in the primary care setting; 2) the patient needs to be referred to hospital care. The patients who may remain in the primary care setting can either need no care or low-complexity care, e.g. medication. The flow of patients is visualised in Fig. 1.

The study population consisted of adult patients (≥18 years) with non-acute and low-complexity cardiology-related health complaints who visited the cardiologist at the PC+ centre between January 1 and December 31, 2015.

### Data collection

Data were collected between January 1 and December 31, 2015 and were extracted from the electronic medical record system of the PC+ centre. The data consisted of all patients referred by the GPs to the PC+ centre during the above-mentioned time period, including information about the patient characteristics (i.e. age and gender), the referral characteristics (i.e. the referral indication and reason for referral both given by the GP), and the advice of the cardiologist regarding follow-up after PC+ of each patient (see Table 1). The referral indication categories were based on the standardised cardiology referral procedure that was set up by the cardiologists and the regional care group. The categories of the variable reason for referral were based on previous research by Van Hoof et al. [23].

**Table 1:** Overview of the variables and categories

Variables	Categories
Gender	Male Female
Age	**–**
Referral indication given by the general practitioner*	Heart palpitations Heart murmur Cardiac screening Suspected arrhythmia Atypical chest pain Reduced exercise capacity Collapse Abnormal ECG Dyspnoea Suspected heart failure Suspected coronary sclerosis Analyses of atrial fibrillation Stable Angina Pectoris
Reason for referral given by the general practitioner*	To reassure the patient Upon patient request Upon specialist advice or request To exclude disease Screening of unclear pathology Checking the unknown To confirm disease
Advice given by the cardiologist after PC+	Follow-up in primary care setting Follow-up in hospital care setting

*Categories are included as separate variables in the analysis

### Data analysis

Descriptive statistics were computed to provide information about the study population. Data were described using absolute counts and percentages for categorical variables, and means and standard deviations for continuous variables. Statistical model assumptions were examined before conducting further analyses.

Logistic regression analyses were performed to investigate the predictive value of the independent variables on the dependent variable. The dependent variable, the advice of the cardiologist regarding the follow-up of the patient, consists of two possibilities: 1) follow-up in primary care setting; 2) follow-up in hospital care setting. Gender, age, referral indication (13 categories) and reason for referral (7 categories) are the independent variables. Age was rescaled such that one unit (year) is equal to ten years. The categories of the 13 indications for referral and seven reasons for referral were used as separate variables with dichotomous coding. An overview of the variables is provided in Table 1. Only people with no missing data were included in the analyses.

First, univariate logistic regression analyses were performed to identify the independent predictors of the outcome of PC+ and to perform a pre-selection for the multivariable logistic regression analyses. Odds ratios (ORs), 95% confidence intervals (CIs) and *p*-values were reported. A p-value cut-off point of 0.15 was used to select the candidate variables for the multivariable analyses. This is also called purposeful selection of covariates [24–26].

Second, multivariable logistic regression analyses were performed using a stepwise backward method. Model selection was based on likelihood ratio test statistics. The validity was assessed using a Hosmer-Lemeshow goodness-of-fit Chi-square analysis. Odds ratios (ORs), 95% confidence intervals (CIs) and *p*-values were reported; p-values < 0.05 were considered statistically significant. Analyses were performed using IBM SPSS Statistics, version 24.

## Results

The data extracted from the electronic medical record system resulted in 1681 cases. Of these, 1525 cases were included for analyses as 156 cases contained missing values on the variables referral indication (*n* = 116), reason for referral (*n* = 36) or both (*n* = 4). The patients’ average age was 57.6 years (SD ± 14.6), with a minimum and maximum age of 18 and 97, respectively, and 46.4% of the patients were male. In 23.1% (*n* = 352) of the cases the cardiologist advised referring the patient to hospital care, while in all other cases (*n* = 1173; 76.9%) the cardiologists’ advice was to keep the patient in primary care. See Table 2 for an overview of the descriptive analyses.

**Table 2:** Description of study population (*N* = 1525)

Variables	Mean (SD±) or n (%)
Gender:	
Female	818 (53.6%)
Male	707 (46.4%)
Age	57.6 (±14.6)
Referral indication given by the general practitioner:	
Atypical chest pain	630 (41.3%)
Dyspnoea	202 (13.2%)
Heart palpitations	192 (12.6%)
Abnormal ECG	103 (6.8%)
Cardiac screening	102 (6.7%)
Reduced exercise capacity	67 (4.4%)
Collapse	60 (3.9%)
Stable Angina Pectoris	55 (3.6%)
Suspected arrhythmia	32 (2.1%)
Suspected heart failure	31 (2.0%)
Heart murmur	25 (1.6%)
Suspected coronary sclerosis	16 (1.0%)
Analyses of atrial fibrillation	10 (0.7%)
Reason for referral given by the general practitioner	
To exclude disease	1193 (81.4%)
Screening of unclear pathology	132 (8.7%)
To confirm disease	78 (5.1%)
To reassure the patient	67 (4.4%)
Checking the unknown	29 (1.9%)
Upon patient request	20 (1.3%)
Upon specialist advice or request	6 (0.4%)
Advice given by the cardiologist after PC+:	
Follow-up in primary care setting	1173 (76.9%)
Follow-up in hospital care setting	352 (23.1%)

Table 3 shows the results of the univariate analyses. Gender, age, four referral indications and five reasons for referrals were independent predictors of the advice ‘follow-up in hospital care setting’. The referral indication ‘Stable Angina Pectoris’ was the most predictive variable (OR = 5.000, 95% CI = 2.885–8.666). The reason for referral ‘To confirm disease’ was the strongest predictor of the advice of the cardiologist (OR = 4.786, 95% CI = 3.006–7.618). The variables gender, age, ‘Atypical chest pain’, ‘Dyspnoea’, ‘Heart palpitations’, ‘Abnormal ECG’, ‘Cardiac screening’, ‘Stable Angina Pectoris’, ‘Suspected heart failure’, ‘To exclude disease’, ‘Screening of unclear pathology’, ‘To confirm disease’, ‘Reassurance’ and ‘Checking the unknown’ had *p*-values smaller than the cut-off point of 0.15 and were included in the multivariable logistic regression analysis.

**Table 3:** Univariate association between independent variables and the advice of the cardiologist: follow-up in hospital care setting after PC+

Independent variables	Advice of the cardiologist: follow-up in hospital care setting after PC+^A^		
	OR	*95% CI*	*P-value*
Gender^B^*	1.694	1.332–2.155	< .001
Age^C^*	1.352	1.236–2.478	< .001
Referral indication			
Atypical chest pain*	0.683	0.533–0.876	.003
Dyspnoea*	1.667	1.206–2.304	.002
Heart palpitations *	0.581	0.386–0.875	.009
Abnormal ECG*	1.477	0.952–2.291	.082
Cardiac screening*	0.650	0.380–1.109	.114
Reduced exercise capacity	0.795	0.429–1.474	.466
Collapse	0.919	0.491–1.718	.791
Stable Angina Pectoris*	5.000	2.885–8.666	< .001
Suspected arrhythmia	0.765	0.312–1.874	.558
Suspected heart failure*	1.860	0.882–3.920	.103
Heart murmur	0.631	0.215–1.849	.401
Suspected coronary sclerosis	2.017	0.728–5.588	.177
Analyses of atrial fibrillation	2.236	0.627–7.967	.215
Reason for referral			
To exclude disease*	0.477	0.365–0.623	< .001
Screening of unclear pathology*	2.368	1.633–3.435	< .001
To confirm disease *	4.786	3.006–7.618	< .001
To reassure the patient*	0.149	0.047–0.477	.001
Checking the unknown*	2.400	1.135–5.075	.022
Upon patient request	0.367	0.085–1.588	.180
Upon specialist advice or request	0.666	0.077–5.715	.711

*Note:* ^*A*^ *The advice was coded as: 0 = Follow-up in primary care setting, 1 = Follow-up in hospital care setting setting;* ^*B*^ *Gender was coded as 0 = female and 1 = male;* ^*C*^ Age was rescaled such that one unit is equal to ten years; * Selected variable for multivariable logistic regression analyses. Predictor with a p < .15

Table 4 presents the independent variables that were significant multivariable predictors of the advice of the cardiologist regarding follow-up after PC+. ‘Stable Angina Pectoris’ (OR = 3.360; 95% CI = 1.850–6.100) and ‘To confirm disease’ (OR = 3.807; 95% CI = 2.307–6.284) are the two strongest predictors. The reason for referral ‘To reassure the patient’ (OR = 0.223; 95% CI = 0.069–0.718) is the only significant predictor with an OR < 1. This implies that patients referred with the reason ‘To reassure the patient’ were more likely to get the advice ‘follow-up in primary care setting’ after the PC+ consultation.

**Table 4:** Multivariable logistic regression analysis: significant predictive variables on the advice of the cardiologist: follow-up in hospital care setting after PC+

Independent variables	Advice of the cardiologist: follow-up in hospital care setting after PC+^A^			
	b	OR	*95% CI*	*P-value*
Gender^B^	0.617	1.854	1.437–2.393	< .001
Age^c^	0.228	1.256	1.143–1.381	< .001
Referral indication				
Dyspnoea	0.458	1.581	1.120–2.231	.009
Stable Angina Pectoris	1.203	3.331	1.847–6.009	< .001
Reason for referral				
Screening of unclear pathology	0.845	2.328	1.580–3.430	< .001
To confirm disease	1.282	3.604	2.180–5.959	< .001
To reassure the patient	−1.475	0.229	0.071–0.739	.014

*Note:* ^*A*^ *The advice was coded as: 0 = Follow-up in primary care setting, 1 = Follow-up in hospital care setting;* ^*B*^ *Gender was coded as 0 = female and 1 = male;* ^*C*^ *Age was rescaled such that one unit is equal to ten years*

## Discussion

### Main findings

The study objective was to explore which cardiology-related referral indications and reasons for referral are appropriate for specialist care in PC+ and which are more appropriate for specialist care in the hospital setting. Results showed that significant predictors of the advice ‘follow-up in hospital care setting’ are gender (male), age (per ten years), the referral indications ‘Stable Angina Pectoris’ or ‘Dyspnoea’ and the reasons for referral ‘To confirm disease’ or ‘Screening of unclear pathology’.

### Interpretation of the main findings

Patients with one or more of the significant predictors have a higher probability of being referred to hospital care after a consultation at the PC+ centre. For example, a patient with an age of 30 years with the referral indication ‘Stable Angina Pectoris’ has a probability of 22.4% of getting the advice ‘Follow-up in hospital care setting’, and when in this case the reason for referral is ‘To confirm disease’ the probability is 51.1%, and if this patient is 70 years (instead of 30 years) it is even 72.2%. The reason for referral ‘To reassure the patient’ is a significant predictor of the advice ‘Follow-up in primary care setting’. The probability of these patients getting the advice ‘Follow-up in hospital care setting’ is only 1%.

The aim of PC+ is to substitute specialist care in the hospital setting with specialist care in the primary care setting. To achieve this it is crucial that PC+ is not designed as an intermediate station and thus it is important to select the appropriate patient groups for PC+. However, the results of this observational explorative study based on quantitative data of the PC+ centre need further interpretation. The results provide only insight which patients are more likely to be referred from PC+ to hospital care. This insight can be used as an input and can provide guidance for the healthcare professionals to improve the referral patterns (e.g. list of inclusion criteria for PC+). GPs and cardiologists should deliberate and collaborate to improve the referring procedures in order to enhance the connectivity between the different healthcare providers and the alignment of care. In addition to the characteristics taken into account in this study, more factors will probably influence which patient groups are appropriate for PC+, such as clinical information and specialist characteristics (e.g. level of expertise, experience and confidence). Hence, further research is needed to find out more precisely which patients are appropriate for PC+ and which patients should be excluded from PC+.

### Reflection with existing literature

Previous studies have shown that outreach clinics could lead to increased healthcare costs [15, 27]. This is also a pitfall of PC+. Focusing on the appropriate patient groups and preventing PC+ from becoming an intermediate station increase its chances of success. Additionally, previous research showed that GPs experienced difficulty and uncertainty in referring eligible patients to PC+ [18]. Medical specialists sometimes saw patients in PC+ who should have been referred directly to hospital care or who could have been treated by the GP him−/herself [18]. Selecting the appropriate patient groups seemed to be essential for achieving efficient substitution. Moreover, PC+ is related to the concept integrated care as described by Kodner and Spreeuwenberg [16]. Intensifying the communication and collaboration between GPs and medical specialists in order to connect and align multiple services, providers and settings is one of its core features. Additionally, it is aimed at enhancing population health, quality of care as experienced by patients and reducing the number of unnecessary and inappropriate referrals in order to achieve efficiency. Hence, to improve the referral procedures it is recommended that GPs and cardiologists deliberate and collaborate. The patient groups that have a significantly higher probability of receiving the advice ‘Follow-up in hospital care setting’ should not be referred first to PC+. Additionally, they should deliberate about the patients who are referred with the reason ‘To reassure the patient’. Previous research indicated that perceived medical need is the strongest predictor but not the only predictor of GPs’ behaviour: perceived pressure from patients is also a strong independent predictor of GPs’ behaviour that affects the referral behaviour [28, 29]. It is plausible that some of the patients with the reason for referral ‘To reassure the patient’ were not referred to PC+ based on a medical need but on perceived pressure from the patients. With reference to efficiency, the results indicate that GPs should refer patients with a medical need and as few patients as possible with only a ‘To reassure the patient’ need. The strengthened collaboration and intensified communication generates knowledge transfer between the GPs and cardiologists. In the long term it is expected that this will induce a learning-effect for the GPs, which will probably result in less inappropriate referrals.

### Strengths and limitations

This study used quantitative data extracted from the electronic medical record system of a cardiology PC+ centre. The study is based on a large sample size, namely on almost all referrals to the centre over one year. The results provide insight into which patients are less appropriate for PC+. Eventually this will lead to an increased number of appropriate referrals and fewer inappropriate referrals, in order to contribute to containing the rising healthcare costs.

A limitation of this study concerns the generalizability of the findings. The findings are context-bound, and generalizability to other countries (e.g. countries where GPs do not have a gatekeeping role), specialties (as this study is focused on cardiology) and/or other PC+ models is limited.

### Future research

The results of this study are based on traditional statistical analyses providing insight into the appropriate patient groups for cardiology PC+ centres. This research is limited to quantitative data, and complementary (qualitative) research may be helpful for interpreting the findings in more detail.

The results showed a gender difference in the probability of being referred to specialist care in the hospital setting after a consultation at PC+; male patients have a significantly higher probability. This gender difference should be further investigated because previous studies demonstrated that the risk of heart disease in women has been underestimated. The awareness of the cardiovascular disease health risk in women among healthcare providers is relatively low and women are often misunderstood in terms of their symptoms [30, 31].

The results of this study can provide guidance for further research. To be better able to select the appropriate patient groups for PC+ it is recommend to investigate the influence of other factors and characteristics, and to perform complementary qualitative research to interpret and clarify the quantitative results. Future research could, for example, focus on the specialist characteristics (e.g. level of expertise, experience and confidence) and it should also include clinical outcomes (e.g. including follow-up data after a consult at the PC+ centre).

Additionally, regarding the cost-effectiveness of this PC+ intervention it is supposed that the introduction of PC+ will lead to reduced healthcare costs by lower costs in PC+ than in hospital care due to lower overhead costs, fewer referrals to hospital care, and less use of additional hospital-based services. Future research will take into account the effects on the health of the population, patients’ experiences of care and healthcare costs and will specifically focus on the cost-effectiveness of PC+ [17].

## Conclusions

Although the success of PC+ does not only depend on selecting the appropriate patient groups, this is important for achieving efficiency. The results of this study can be used to improve the referral pattern and to enhance the connectivity and alignment of care. Patients with the referral indication ‘Stable Angina Pectoris’ or ‘Dyspnoea’, and reason for referral ‘To confirm disease’ or ‘Screening of unclear pathology’, seem to be more appropriate for specialist care in the hospital setting instead of PC+. Additionally, the reason for referral ‘To reassure the patient’ is a significant predictor of the advice ‘Follow-up in primary care setting’.

## Abbreviations

GP: General practitioner
PC+: Primary Care Plus
